# Global and domain-specific cognitive intraindividual variability associations with neurodegenerative diagnoses and postmortem pathologies

**DOI:** 10.1186/s13195-026-02041-4

**Published:** 2026-04-21

**Authors:** Caitlin M. Terao, Fareshte Erani, Uriel Urias, Francesca Lopez, Fini Chang, Sarah J. Banks, Paul E. Gilbert, David G. Coughlin, Katherine J. Bangen, Kelsey R. Thomas

**Affiliations:** 1 San Diego State University/University of California, San Diego Joint Doctoral Program in Clinical Psychology, 6363 Alvarado Ct, San Diego, CA 92120 USA; 2 Department of Psychiatry, University of California San Diego, 9500 Gilman Dr, La Jolla, CA 92093 USA; 3 Department of Psychology, San Diego State University, 5500 Campanile Dr, San Diego, CA 92182 USA; 4 VA San Diego Healthcare System, 3350 La Jolla Village Dr, San Diego, CA 92161 USA; 5 Center of Excellence for Stress and Mental Health (CESAMH), VA San Diego Health Care System, 3350 La Jolla Village Dr, San Diego, CA 92161 USA; 6 Department of Neurosciences, University of California San Diego, 9500 Gilman Dr, La Jolla, La Jolla, CA 92093 USA

**Keywords:** IIV, Neuropsychology, Dementia, Pathology, Progression

## Abstract

**Background:**

Cognitive intraindividual variability (IIV) or within-person variation in neuropsychological test performance relates to in vivo biomarkers of neurodegeneration and progression to mild cognitive impairment (MCI) and dementia. The current study aimed to explore longitudinal associations between global and domain-specific IIV with cognitive status and neuropathological findings at autopsy, and whether these associations differed by sex.

**Methods:**

The sample included 20,715 older adults from the National Alzheimer’s Coordinating Center (NACC) who completed cognitive testing as part of the Uniform Data Set. Participants were cognitively unimpaired at Visit 1. Baseline neuropsychological data was used to calculate coefficient of variation IIV (intraindividual standard deviation/mean performance) for global, executive, language, and memory domains. Global and domain-specific IIV associations with final visit cognitive status, etiological diagnosis, and progression to MCI/dementia were examined. Associations between each IIV score and neuropathological data were examined for participants with available autopsy data (*n*s = 1184–1717). Secondary models explored sex by IIV interactions.

**Results:**

Greater IIV (i.e., more variability) in global and specific domains related to worse cognitive status at final visit and final etiological diagnosis. Across all IIV domains, higher IIV related to greater risk of progressing to MCI/dementia. Higher global IIV related to greater postmortem burden of amyloid-β plaques, neurofibrillary tangles (NFT), AD neuropathologic change (ADNC) scores, hippocampal atrophy, substantia nigra neuron loss, hypopigmentation in the locus coeruleus, and cerebral amyloid angiopathy (CAA). Domain-specific effects included: higher executive IIV related to greater NFT, neuritic plaques, ADNC scores, and CAA; and higher memory IIV related to greater neuritic plaques and NFT. There were no significant interactions of sex and IIV.

**Conclusions:**

Among cognitively unimpaired older adults, global IIV was a sensitive but non-specific marker associated with cognitive decline and neuropathology at autopsy. Domain-specific IIVs were also associated with cognitive progression but offered more specificity in neuropathologic outcomes. Associations were largely consistent across sexes, indicating IIV is robust to sex-specific effects. Findings highlight IIV as sensitive to long-term cognitive health and neuropathological burden and may improve early risk stratification for dementia.

**Supplementary Information:**

The online version contains supplementary material available at 10.1186/s13195-026-02041-4.

## Background

Cognitive intraindividual variability (IIV) refers to within-person variation in neuropsychological test performance and is sensitive to early cognitive changes in Alzheimer’s disease and related dementias (ADRD) [1, 2]. IIV has been defined as dispersion (i.e., variability across different neuropsychological measures at a single time point) and inconsistency (i.e., variability in performance across trials of a single task) [3, 4]. IIV reflects the magnitude of variation, so it does not differentiate positive (i.e., above average performance) from negative (i.e., below average performance) variation. This is particularly relevant among cognitively unimpaired individuals who are more likely to have higher IIV due to positive variation. Coefficient of variation (CoV) is a measure of dispersion that accounts for this possibility by including mean performance within the calculation of IIV. By incorporating mean performance, CoV helps differentiate non-normative variation among cognitively unimpaired older adults, potentially improving early detection of subtle cognitive decline [5]. 

IIV has an established link with key ADRD outcomes. IIV relates to biomarkers of neurodegeneration including regional brain atrophy, cerebral blood flow, and APOE ε4 status [6–11]. IIV is also associated with cognitive functioning, functional abilities, and progression to mild cognitive impairment (MCI) and ADRD [8, 12–14]. A meta-analysis of 35 studies found a moderate effect of IIV in distinguishing cognitively unimpaired older adults from individuals with MCI and AD, with IIV increasing along with severity of cognitive impairment [1]. Additionally, a recent review article by our group, including 44 articles, found strong evidence for IIV associations with ADRD outcomes and in vivo biomarker burden even after adjusting for mean cognitive performance. Specifically, with respect to biomarkers, higher IIV related to greater neurofibrillary tangle (NFT) burden, amyloid-β positivity, poorer white matter integrity, and lower hippocampal, entorhinal, posterior cingulate, precuneus, and superior frontal gyrus brain volumes [2]. 

While global IIV (i.e., variation across multiple cognitive domains) research is more prevalent, limited previous work has examined domain-specific IIV (i.e., variation within a single cognitive domain) and associations with ADRD outcomes. Executive IIV predicts long-term cognitive and functional outcomes among cognitively unimpaired older adults, adults with MCI, and older adults with AD; [14] however, other studies report no associations between domain-specific IIV (i.e., attention, language, visuospatial, memory, and executive function) and longitudinal cognitive status or decline [15]. In terms of differentiating diagnostic groups, one study found that memory IIV but not executive IIV differentiated individuals with AD, MCI, and no cognitive impairment [12], whereas another study found that executive IIV, but not global IIV, distinguished cognitively unimpaired older adults from those with MCI [16]. Past research on domain-specific IIV and neuroimaging outcomes among cognitively unimpaired older adults has shown that higher memory IIV, but not global IIV or executive IIV, related to lower hippocampal volumes [17], whereas higher global IIV, but not memory IIV or executive IIV, was associated with poorer white matter integrity [18]. To address gaps in prior research, we examined how global and domain-specific IIV may differentially signal long-term ADRD outcomes including cognitive status and progression to MCI/dementia in a large, well-characterized sample. We will further extend past research, which has largely focused on neuroimaging and biomarkers, to examine IIV associations with postmortem neuropathologic burden.

Notably, most studies of IIV in the context of ADRD have adjusted for sex, therefore masking potential sex-specific relationships. Previous research on sex and IIV has found that dispersion and inconsistency IIV are higher in females compared to males, yet other work has found no sex differences in these metrics [19–22]. Prior work has also shown that sex may moderate IIV associations with physical activity and biological vitality outcomes, such that these relationships are stronger in males relative to females [21, 23]. Past studies exploring IIV associations with consideration for sex have been limited and inconsistent, warranting further research to clarify sex-specific relationships between IIV and ADRD outcomes.

Our primary aim was to examine associations among cognitively unimpaired older adults of global and domain-specific cognitive IIV with longitudinal cognitive status/diagnosis, progression to MCI/dementia, and neuropathological burden at autopsy. As a secondary aim, we examined whether these associations differ by sex. We hypothesized that global IIV would be associated with longitudinal cognitive status/diagnosis, progression to MCI/dementia, and broad measures of neuropathological burden, whereas measures of domain-specific IIV may show more unique associations with these outcomes (e.g., memory IIV associations with AD, executive IIV associations with cerebrovascular disease). We also hypothesized that associations with global and domain-specific IIV would differ by sex, such that the observed relationships would be stronger in males compared to females.

## Methods

### Participants

The current sample included National Alzheimer’s Coordinating Center (NACC) participants who completed neuropsychological measures as part of the Uniform Data Set (UDS). Participants were recruited from 46 Alzheimer’s Disease Research Center (ADRC) study sites between 2005 and 2025. Detailed information regarding NACC UDS study samples and protocol are available (naccdata.org). At the time of recruitment, NACC participants had a stable health status with no history of major stroke, neurologic disorders, severe psychiatric illness, substance abuse, or learning disability. The present study included participants who at their first visit (*n* = 54,025) were cognitively unimpaired (*n* = 22,242), over the age of 50 years old (*n* = 21,354), and had available neuropsychological data (final *n* = 20,715).

### Materials

#### Cognitive intraindividual variability

Visit one neuropsychological data, described previously [24], was used to calculate CoV IIV for global, executive, language, and memory domains. CoV was calculated for each participant as the intraindividual standard deviation (ISD) across neuropsychological scores divided by their mean performance on those same measures, with higher scores indicating greater performance variability relative to their mean performance [2]. For global IIV, mean performance included all measures, whereas domain-specific mean scores were calculated for each IIV domain (i.e., ISD/M on the same measures). Before calculating each IIV measure, raw neuropsychological scores were converted to a T-score, and relevant scores were reverse-coded so that higher scores consistently indicated better performance.

Global IIV included measures of executive function, language, and memory. Specific scores included total scores for Logical Memory (LM) immediate recall, LM delayed recall, Benson Complex Figure Test (BCFT) delayed recall, BCFT Copy, Digit Span (DS) Forward, DS Backward, Semantic Fluency (Vegetables and Animals), Phonemic Fluency (L and F words), Boston Naming Test (BNT) 30, Multilingual Naming Test (MINT), Number Span Test (NST) Forward, NST Backward, Digit Symbol Substitution Test (DSST), Craft Story 21 Test (CST) immediate paraphrased recall, CST delayed paraphrased recall, and time to completion for Trail Making Test (TMT) Part A and TMT Part B. Given the use of data from UDS 1.0–3.0, participants did not have all listed measures available; see Supplementary Table 1 for the full breakdown. Domain-specific IIV estimates required at least two contributing tests; otherwise, values were coded as missing. Of note, 99.98% of participants had at least two scores available for each of the domain-specific IIV scores described below, minimizing concerns regarding instability due to differing test counts. Variation in the number of contributing tests primarily reflects structural differences across UDS versions rather than selective participant-level missingness.

Domain-specific IIV included scores from the global IIV measure within the cognitive domains of executive function, language, and memory. Executive IIV included total scores for DS Forward, DS Backward, NST Forward, NST Backward, and DSST as well as time to completion for TMT Part A and TMT Part B. Language IIV included total scores for Semantic Fluency (Vegetables and Animals), Phonemic Fluency (L and F words), BNT 30, and MINT. Memory IIV included total scores for LM immediate recall, LM delayed recall, BCFT delayed recall, CST immediate paraphrased recall, and CST delayed paraphrased recall.

#### Cognitive status and etiological diagnosis

NACC cognitive status, determined at each visit via team consensus or a single physician [25], is classified as normal cognition, impaired-not-MCI (i.e., impaired cognition that does not meet criteria for MCI), MCI, or dementia. Dementia diagnoses were in line with the National Institute on Aging-Alzheimer’s Association (NIA-AA) diagnostic framework as well as the latest diagnostic criteria for non-ADRD dementias [25, 26]. Etiological diagnoses were based on the consensus conference determination of the primary and probable cause of observed cognitive impairment at each participant’s final study visit, consistent with a previously described operational approach to coding etiological diagnoses in NACC [27]. Major neurodegenerative conditions included AD, Lewy body disease (LBD), Parkinson’s disease, atypical Parkinsonism syndromes (e.g., multiple system atrophy, progressive supranuclear palsy, corticobasal degeneration), frontotemporal dementia (FTD; including FTD with motor neuron disease, behavioral variant FTD, and FTD not otherwise specified), primary progressive aphasias (PPA), and vascular (e.g., vascular brain injury and vascular dementia). Here, we were specifically interested in the cognitive status and etiological diagnosis assigned at each participant’s final visit.

#### Neuropathology

Neuropathologic assessment was available for a subset of participants, the methods and operationalization of which have been previously described [25, 27]. Given the exploratory aims, all available neuropathological outcomes were included, except those with insufficient variability (e.g., TDP-43 pathology). Specific measures of interest included NIA-AA Alzheimer’s disease neuropathologic change (ADNC; included: not AD, low, intermediate, high), neocortical neuritic plaque density (CERAD; included: none, sparse, moderate, frequent), Thal phase for amyloid-β plaques (i.e., both diffuse and dense-core; Thal; included phases: 0–2, 3, and 4–5), NFT pathology severity (Braak; included stages: 0-II, III-IV, V-VI), Lewy body pathology (subtypes included: none, brainstem/olfactory/amygdala/unspecified, limbic/neocortical) [28], frontotemporal lobar degeneration with tau pathology or other tauopathy (FTLD-Tau; included: present, absent), arteriolosclerosis (included: none, mild, moderate, severe), atherosclerosis (Circle of Willis; included: none, mild, moderate, severe), cerebral amyloid angiopathy (CAA; included: none, mild, moderate, severe), infarcts and lacunes (included: present, absent), microinfarcts (included: present, absent), white matter rarefaction (included: none, mild, moderate, severe), cerebral atrophy (included: none, mild, moderate, severe), hippocampal atrophy (included: none, mild, moderate/severe), locus coeruleus hypopigmentation (included: none, mild, moderate, severe), substantia nigra hypopigmentation (included: none, mild, moderate/severe), and substantia nigra neuron loss (included: none, mild, moderate/severe).

We included measures of ADRD severity (i.e., CERAD, Thal, Braak), and ADRD primary pathology (i.e., ADNC) based on NIA-AA consensus criteria, with Thal phase 4–5, Braak stage V-VI, and moderate-to-severe neuritic plaque density indicating high ADNC [28, 29]. We also included neuropathological outcomes indicative of widespread neurodegenerative and vascular pathologies, including proteinopathies (i.e., Lewy body, FTLD-Tau), cerebrovascular disease (i.e., arteriolosclerosis, atherosclerosis, CAA, infarcts and lacunes, microinfarcts, white matter rarefaction), and gross markers of neurodegeneration (i.e., cerebral atrophy, hippocampal atrophy, substantia nigra hypopigmentation, locus coeruleus hypopigmentation, substantia nigra neuron loss).

### Analyses

Descriptive and clinical characteristics by sex were explored using linear regression models and Chi-square tests. Ordinal regression modeling examined global and domain-specific IIV associations as the independent variable with final visit cognitive status as the dependent variable, adjusting for sex (male/female), age, years of education, years since visit one, and race (White, Black, and other/unknown due to low numbers). Odds ratios (ORs) and corresponding 95% confidence intervals (CIs) were calculated by exponentiating model coefficients and using profile likelihood estimation. Multinomial logistic regression modeling examined global and domain-specific IIV associations as the independent variable with final visit etiological diagnosis as the dependent variable, adjusting for sex, age, years of education, years since visit one, and race. ORs and Wald-based 95% CIs were obtained by exponentiating the model coefficients. Cox proportional-hazard models examined longitudinal progression to MCI/dementia (i.e., years from visit one until their first observed transition to consensus diagnosis of MCI or dementia) by global and domain-specific IIV tertiles (i.e., each IIV metric was divided into tertiles of low, average, and high IIV based on the sample distribution), adjusting for sex, age, years of education, and race. The IIV tertiles were defined using the 33rd and 66th percentiles of each IIV metric, resulting in low variability ($\:\le\:$33 percentile), average variability (33–66 percentile), and high variability (>66 percentile) IIV groups. Sensitivity analyses included Cox models weighted by inverse probability of censoring weights derived from a separate model predicting censoring as a function of demographic covariates, and Fine–Gray subdistribution hazard models treating death prior to conversion as a competing risk. There were no differences in variable significance, including pairwise comparisons, so the unweighted Cox models are reported here. Kaplan-Meier curves were used to depict the rate of progression to cognitive impairment over time. Ordinal and binomial logistic regressions examined associations between global and domain-specific IIV with neuropathological outcomes, adjusting for sex, age, years of education, race, and years since visit one (i.e., years from visit one to autopsy). For all listed ordinal and binomial models, secondary models explored sex by IIV interactions, adjusting for the same covariates. Benjamini-Hochberg False Discovery Rate Method (FDR) was applied to adjust for multiple comparisons within each set of analyses for cognitive status, neuropathological burden, and sex interactions [30]. All analyses were conducted in R (version 4.4.0).

## Results

The final analytic sample included 20,715 NACC participants who were cognitively unimpaired at their first visit. Participants were on average 70.7 years old (*SD* = 8.9, *Range* = 50–104), 76.3% White, and had an average education of 15.9 years (*SD* = 3.0, *Range* = 0–30). Participants had an average of 4.6 years of follow-up data available (*SD* = 4.7, *Range* = 0-19.2). In terms of sex differences, females were younger, less educated, more racially diverse, and lived longer relative to males within the sample. Sex differences in final clinical diagnosis were statistically significant but small in magnitude, with similar AD prevalence between females and males, slightly fewer males classified as cognitively unimpaired, and a higher prevalence of LBD among males. Additionally, females had lower language IIV and higher executive IIV and global IIV compared to males. Given the demographic differences observed between males and females, follow-up analyses examined IIV differences by sex while adjusting for age, education, and race. Results showed that females had higher global IIV (*p*<.001) and lower language IIV (*p*<.001) compared to males, but that there was no sex difference in executive IIV after demographic adjustment (*p*=.434). See Table 1 for full sample characteristics by sex.

Descriptive characteristics for participants with any available neuropathological outcome data (*n* = 1719) were compared to those without available autopsy data (*n* = 18996). Briefly, those with available autopsy data were older, disproportionately White, and more likely to have a dementia diagnosis at their final visit. In terms of IIV, the autopsy subset had higher global IIV and language IIV as well as lower executive IIV and memory IIV compared to those without available autopsy data. See Supplementary Table 2 for full results. Due to low counts for Parkinson’s disease (*n* = 6), atypical Parkinsonism syndromes (*n* = 11), FTD (*n* = 38), and PPA (*n* = 4), these diagnostic categories as well as individuals with dementia who did not meet criteria for any of the listed major neurodegenerative conditions (*n* = 67) were combined into an “other dementia” category (*n* = 126). The clinical heterogeneity of this group limits interpretability; therefore, we present results for this category in the Supplementary Material.

**Table 1:** Sample characteristics of the total sample and by sex

	Total (*n* = 20715)	Females (*n* = 13647)	Males (*n* = 7068)	Test for Group Differences
Age^1^, M(SD)	70.65 (8.91)	70.32 (8.90)	71.27 (8.89)	*p*<.001
Education, *M*(*SD*)	15.85 (2.95)	15.57 (2.91)	16.39 (2.97)	*p*<.001
Race, *n* (%)				*p*<.001
White	15,802 (76.28)	9975 (73.09)	5827 (82.44)	
Black	3697 (17.85)	2820 (20.66)	877 (12.40)	
Asian	593 (2.86)	396 (2.90)	197 (2.79)	
AIAN	212 (1.02)	167 (1.22)	45 (0.64)	
NHPI	18 (0.09)	13 (0.01)	5 (0.07)	
Other	258 (1.25)	184 (1.35)	74 (1.05)	
Unknown	135 (0.65)	92 (0.77)	43 (0.61)	
Final Diagnosis, *n* (%)				*p*<.001
CU	17,063 (82.37)	11,354 (83.20)	5709 (80.77)	
Impaired-not-MCI	575 (2.78)	362 (2.65)	213 (3.01)	
MCI	453 (2.19)	288 (2.11)	165 (2.33)	
AD	2035 (9.82)	1324 (9.70)	711 (10.06)	
Vascular	295 (1.42)	185 (1.36)	110 (1.56)	
LBD	168 (0.81)	64 (0.47)	104 (1.47)	
Other	126 (0.61)	70 (0.51)	56 (0.80)	
Years Death, *M*(*SD*)	8.13 (4.62)	8.44 (4.67)	7.66 (4.49)	*p*<.001
IIV Global	0.89 (0.29)	0.90 (0.29)	0.88 (0.28)	*p*<.001
IIV Language	0.37 (0.14)	0.35 (0.13)	0.42 (0.14)	*p*<.001
IIV Executive	0.99 (0.24)	0.99 (0.24)	0.97 (0.23)	*p*<.001
IIV Memory	0.17 (0.14)	0.17 (0.14)	0.17 (0.14)	*p*=.209

*AIAN* American Indian or Alaska Native, *NHPI* Native Hawaiian or Other Pacific Islander, *CU* Cognitively unimpaired, *AD* Alzheimer’s disease, *MCI* Mild cognitive impairment

^1^Age at baseline visit

### Cognitive status

Ordinal regression modeling examined global and domain-specific IIV associations with final visit cognitive status (i.e., cognitively unimpaired, impaired-not-MCI, MCI, or dementia), adjusting for covariates. Higher global IIV was significantly associated with worse final visit cognitive status, with an OR of 4.1 (95% CI: 3.5–4.7, *p*<.001), indicating a 4.1-fold increase in odds for more advanced cognitive impairment stage per unit increase in global IIV. Greater language IIV (OR = 5.0, 95% CI: 3.7–6.7, *p*<.001), memory IIV (OR = 2.2, 95% CI:1.8–2.8, *p*<.001), and executive IIV (OR = 3.0, 95% CI:2.5–3.6, *p*<.001) each showed significant associations with worse cognitive status at final visit.

**Fig. 1:**
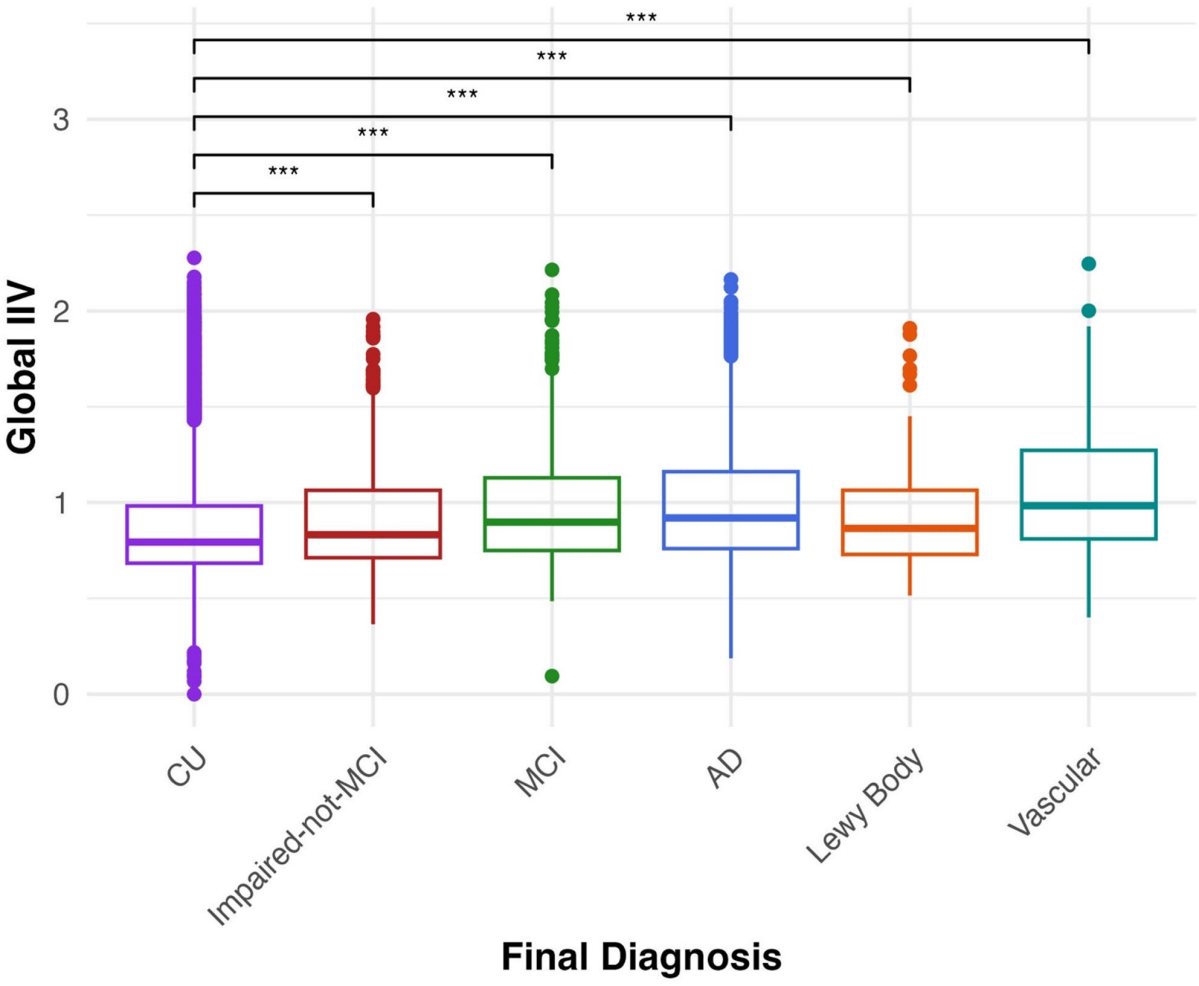
Global IIV by etiological diagnosis

Multinomial logistic regression modeling demonstrated global and domain-specific IIV associations with final visit etiological diagnosis, adjusting for covariates, such that higher IIV was associated with a greater likelihood of cognitive impairment. Participants with higher global IIV had greater odds of being classified as vascular (OR = 5.5, 95% CI: 3.8–8.2, *p*<.001), AD (OR = 4.8, 95% CI: 4.0-5.7, *p*<.001), LBD (OR = 4.4, 95% CI: 2.5–7.9, *p*<.001), MCI (OR = 3.2, 95% CI: 2.3–4.4, *p*<.001), and impaired-not-MCI (OR = 2.2, 95% CI: 1.6-3.0, *p*<.001) relative to participants who remained cognitively unimpaired. See Fig. 1.

In terms of language IIV, participants who at their last visit were classified as AD (OR = 5.9, 95% CI: 4.0-8.7, *p*<.001), LBD (OR = 5.7, 95% CI: 1.8–18.5, *p*=.006), vascular (OR = 5.7, 95% CI: 2.4–13.6, *p*<.001), and MCI (OR = 5.4, 95% CI: 2.7–10.9, *p*<.001) demonstrated greater variability relative to participants who remained cognitively unimpaired. There was no significant difference in language IIV between cognitively unimpaired participants and those classified as impaired-not-MCI.

For memory IIV, participants who at their final visit were classified as AD demonstrated greater variability relative to participants who remained cognitively unimpaired (OR = 3.8, 95% CI: 2.8–5.1, *p*<.001). There were no significant differences in memory IIV between cognitively unimpaired participants and those classified as impaired-not-MCI, MCI, LBD, and vascular.

For executive IIV, participants who at their final visit were classified as vascular (OR = 5.1, 95% CI: 2.9–8.7, *p*<.001), LBD (OR = 4.0, 95% CI: 1.9–8.7, *p*<.001), AD (OR = 3.3, 95% CI: 2.6–4.2, *p*<.001), MCI (OR = 2.6, 95% CI: 1.7–4.1, *p*<.001), and impaired-not-MCI (OR = 1.6, 95% CI: 1.1–2.4, *p*=.038) demonstrated greater variability relative to participants who remained cognitively unimpaired.

**Fig. 2:**
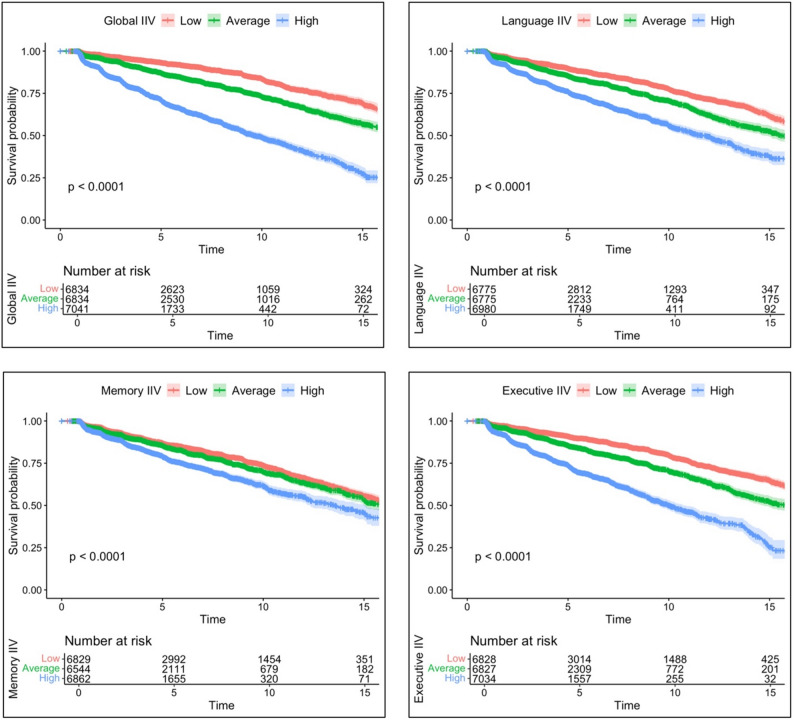
Progression to MCI/Dementia by IIV Tertile. The model adjusting for sex, age, years of education, and race.

By their final visit, 3,077 participants progressed from cognitively unimpaired to MCI/dementia (impaired-not-MCI was not considered as progression within these analyses). Cox proportional-hazard models examined longitudinal progression to MCI/dementia by global and domain-specific IIV tertiles, adjusting for covariates. Pairwise comparisons demonstrated a stepwise increase in the risk of progression to cognitive impairment with higher global and domain-specific IIV. Participants in the highest global IIV group had a greater risk of progressing to cognitive impairment than the lowest (HR = 3.8, 95% CI: 3.3–4.3, *p*<.001) and average (HR = 2.3, 95% CI: 2.1–2.6, *p*<.001) global IIV groups. Additionally, the average global IIV group showed a higher risk of progressing to cognitive impairment than the lowest global IIV group (HR = 1.6, 95% CI: 1.4–1.9, *p*<.001). Participants in the highest language IIV group had a greater risk of progressing to cognitive impairment than the lowest (HR = 2.2, 95% CI: 1.9–2.4, *p*<.001) and average (HR = 1.6, CI: 1.4–1.8, *p*<.001) language IIV groups. Additionally, the average language IIV group showed a higher risk of progressing to cognitive impairment than the lowest language IIV group (HR = 1.4, 95% CI: 1.2–1.5, *p*<.001). Participants in the highest memory IIV group had a greater risk of progressing to cognitive impairment than the lowest (HR = 1.7, 95% CI: 1.5–1.9, *p*<.001) and average (HR = 1.4, 95% CI: 1.3–1.6, *p*<.001) memory IIV groups. Additionally, the average memory IIV group showed a higher risk of progressing to cognitive impairment than the lowest memory IIV group (HR = 1.2, 95% CI: 1.1–1.3, *p*<.001). Participants in the highest executive IIV group had a greater risk of progressing to cognitive impairment than the lowest (HR = 2.9, 95% CI: 2.6–3.3, *p*<.001) and average (HR = 1.9, 95% CI: 1.7–2.2, *p*<.001) executive IIV groups. Additionally, the average executive IIV group showed a higher risk of progressing to cognitive impairment than the lowest executive IIV group (HR = 1.5, 95% CI: 1.4–1.7, *p*<.001). See Fig. 2 for Kaplan Meier curves.

### Neuropathology

Ordinal and binomial logistic regressions examined associations between global and domain-specific IIV with neuropathological outcomes, adjusting for covariates. Results are presented in Table 2. For neuropathologic measures of ADRD severity, higher global and executive IIV were significantly associated with greater ADNC. Higher global, memory, and executive IIV were significantly related to both higher CERAD scores and Braak stages. Additionally, higher global IIV was significantly associated with higher Thal phases. In terms of neuropathological outcomes indicative of cerebrovascular disease, higher global and executive IIV related to greater CAA. For neuropathological outcomes related to gross markers of neurodegenerations, higher global IIV was significantly associated with greater hippocampal atrophy, locus coeruleus hypopigmentation, and substantia nigra neuron loss. There were no other significant global or domain-specific IIV associations with additional neuropathological outcomes, including additional measures of cerebrovascular disease and neurodegeneration.

**Table 2:** IIV indices and neuropathology

	OR	95% CI		p_BH_
		LL	UL	
ADNC (*n*=1232)				
Global IIV	1.71	1.13	2.60	.025
Language IIV	1.07	0.49	2.36	.861
Memory IIV	1.32	0.58	2.99	.679
Executive IIV	1.83	1.14	2.93	.025
CERAD (*n*=1717)				
Global IIV	1.67	1.20	2.31	.007
Language IIV	0.87	0.44	1.72	.689
Memory IIV	2.73	1.35	5.54	.007
Executive IIV	1.69	1.19	2.41	.007
Thal (*n*=1242)				
Global IIV	1.77	1.14	2.74	.044
Language IIV	1.25	0.55	2.83	.597
Memory IIV	1.32	0.56	3.17	.597
Executive IIV	1.61	0.98	2.66	.126
Braak (*n*=1700)				
Global IIV	1.68	1.19	2.36	.006
Language IIV	1.11	0.55	2.22	.768
Memory IIV	3.18	1.51	6.76	.006
Executive IIV	1.65	1.13	2.39	.012
Lewy Bodies (*n*=1714)				
Global IIV	1.07	0.68	1.67	.872
Language IIV	0.70	0.29	1.69	.872
Memory IIV	1.09	0.42	2.71	.872
Executive IIV	0.96	0.59	1.58	.872
FTLD-Tau (*n*=1227)				
Global IIV	1.18	0.64	2.14	.651
Language IIV	1.65	0.53	5.01	.651
Memory IIV	1.31	0.39	4.07	.651
Executive IIV	1.51	0.75	3.04	.651
Atherosclerosis (*n*=1709)				
Global IIV	1.14	0.82	1.59	.724
Language IIV	0.76	0.39	1.48	.724
Memory IIV	0.80	0.39	1.64	.724
Executive IIV	0.95	0.66	1.37	.782
Arteriolosclerosis (*n*=1542)				
Global IIV	1.39	0.98	1.98	.139
Language IIV	1.94	0.95	4.00	.139
Memory IIV	1.25	0.59	2.67	.757
Executive IIV	1.00	0.68	1.47	.987
Cerebral amyloid angiopathy (*n*=1694)				
Global IIV	1.80	1.27	2.54	.004
Language IIV	1.61	0.81	3.21	.177
Memory IIV	1.79	0.85	3.73	.165
Executive IIV	1.78	1.22	2.62	.006
Infarcts and Lacunes (*n*=1714)				
Global IIV	1.03	0.65	1.60	.911
Language IIV	0.74	0.28	1.90	.712
Memory IIV	0.61	0.21	1.67	.695
Executive IIV	0.68	0.42	1.10	.450
Microinfarcts (*n*=1716)				
Global IIV	1.10	0.72	1.66	.811
Language IIV	1.75	0.75	4.09	.778
Memory IIV	0.87	0.35	2.15	.811
Executive IIV	1.06	0.67	1.67	.811
White Matter Rarefaction (*n*=1188)				
Global IIV	1.61	1.04	2.49	.098
Language IIV	2.28	1.00	5.20	.098
Memory IIV	1.47	0.63	3.45	.503
Executive IIV	1.08	0.65	1.78	.777
Substantia Nigra Neuron Loss (*n*=1241)				
Global IIV	1.81	1.16	2.83	.036
Language IIV	1.61	0.70	3.66	.345
Memory IIV	1.16	0.49	2.75	.730
Executive IIV	1.60	0.97	2.66	.140
Substantia Nigra Hypopigmentation (*n*=1230)				
Global IIV	1.33	0.82	2.15	.974
Language IIV	1.05	0.42	2.63	.974
Memory IIV	0.74	0.27	1.91	.974
Executive IIV	1.01	0.59	1.74	.974
Locus Coeruleus Hypopigmentation (*n*=1184)				
Global IIV	1.86	1.17	2.95	.036
Language IIV	1.05	0.44	2.51	.914
Memory IIV	1.11	0.44	2.74	.914
Executive IIV	1.46	0.86	2.51	.322
Cerebral Atrophy (*n*=1202)				
Global IIV	1.47	0.94	2.29	.366
Language IIV	1.39	0.60	3.18	.881
Memory IIV	1.21	0.50	2.93	.883
Executive IIV	1.04	0.63	1.73	.883
Hippocampal Atrophy (*n*=1210)				
Global IIV	1.97	1.27	3.04	.009
Language IIV	1.87	0.81	4.33	.146
Memory IIV	2.04	0.87	4.79	.146
Executive IIV	1.45	0.89	2.38	.146

*OR O*dds ratio, *CI C*onfidence interval, *LL* Lower limit, *UL U*pper limit, *P*_*BH*_ Benjamini-Hochberg-adjusted p-value, *ADNC *Alzheimer’s disease neuropathologic change, *CERAD * Neocortical neuritic plaque density, *Braak * Neurofibrillary tangle pathology severity, *Thal *Thal phase for amyloid plaques, *FTLD-Tau * Frontotemporal lobar degeneration with tau pathology or other tauopathy

### Sex interactions

Secondary analyses explored sex by IIV interactions for all previously listed ordinal and binomial models, adjusting for the same covariates. After FDR corrections were applied, there were no significant sex by IIV interactions in any of the ordinal or binomial regression models (all *p*s>0.05). Prior to FDR correction, there was a significant sex by language IIV interaction for final visit cognitive status, such that the relationship was stronger in males compared to females. Additionally, there was a significant sex by memory IIV interaction in Braak staging, such that this relationship was significant in females but not males. See Supplementary Table 3 for full results and Supplementary Table 4 for follow-up sex-stratified analyses.

## Discussion

In this study of cognitively unimpaired older adults, we calculated global and domain-specific IIV and examined longitudinal associations with cognitive status, cognitive diagnosis, progression to MCI/dementia, and neuropathological outcomes at autopsy. Secondary analyses explored sex-specific relationships between IIV and study outcomes. Higher global and domain-specific IIV (i.e., more variability) were associated with more impaired final cognitive status, final etiological diagnosis, and greater odds of progressing to MCI/dementia. Global IIV was sensitive to cognitive decline and demonstrated broad associations with AD, gross neurodegenerative, and cerebrovascular neuropathological findings. Domain-specific IIVs were also sensitive to cognitive decline but showed more specificity in neuropathological outcomes. Executive IIV was sensitive to AD and CAA neuropathological outcomes, whereas memory IIV was uniquely sensitive to AD neuropathology. Associations were robust to sex-specific effects with IIV, demonstrating comparable utility in males and females across outcomes.

Consistent with prior work, global IIV was associated with cognitive decline and risk of progression to MCI/dementia [31–33]. Extending these findings, higher executive, language, and memory IIV domains were also related to worse final visit cognitive status and greater risk of progression to cognitive impairment. The association between global IIV and risk of progression to MCI/dementia was stronger than the domain-specific associations, which is also in line with previous findings [14]. It is possible that the inclusion of more measures within the calculation of global IIV relative to domain-specific IIV metrics accounts for this difference in relationship strength, as the inclusion of more diverse measures may result in greater reliability [2]. Additionally, global IIV may better capture relative weaknesses across measures (which may start within just one domain or measure) than measures of variability within a specific domain. Regardless, both global and domain-specific IIV demonstrated associations with progression to MCI/dementia that suggest IIV may help predict future risk of cognitive impairment.

While research relating IIV with progression to cognitive impairment and decline has been increasingly prevalent, limited research has examined IIV associations with specific neurodegenerative diagnoses. Here, we demonstrated that cognitively unimpaired older adults’ global and domain-specific IIV related to their etiological clinical diagnosis at their final follow-up visit. Relative to cognitively unimpaired participants, participants classified as impaired-not-MCI, MCI, AD, LBD, or vascular all had higher global IIV. Results were largely consistent across language IIV and executive IIV, despite one exception (i.e., no difference with impaired-not-MCI in language IIV), attributed to the small sample size and heterogeneity of the impaired-not-MCI diagnostic category. Memory IIV was uniquely higher in participants with AD, suggesting it may be a behavioral marker specific to AD pathology, while global, executive, language IIV may reflect broader neurodegenerative processes.

Higher global IIV demonstrated diverse associations with neuropathological outcomes, including greater ADNC scores, CERAD scores, Thal phases, Braak stages, CAA, hippocampal atrophy, locus coeruleus hypopigmentation, and substantia nigra neuron loss. Consistent with past work linking global IIV to ADRD biomarkers [6, 34, 35], the current study extends evidence of its relationships across broad postmortem metrics. Previous studies have demonstrated associations between global IIV and neuroimaging markers of cerebrovascular disease (i.e., white matter hyperintensities, cerebral blood flow, and white matter integrity) among cognitively unimpaired individuals [9, 18, 36], and the current study builds on this by demonstrating a relationship with postmortem CAA severity. One study among American Indians with MCI found that hippocampal volumes related to memory IIV but not global or executive IIV [17], whereas the current study showed greater hippocampal atrophy associated with higher global IIV only. The discrepancy between this previous study and our current findings may reflect differences in sample characteristics (e.g., ethnoracial, recruitment approaches, cognitive status), operationalization of hippocampal atrophy (i.e., in vivo and cross-sectional vs. autopsy and longitudinal), cognitive measures included in IIV, and/or other differences in study design. Higher global IIV also related to greater locus coeruleus hypopigmentation and substantia nigra neuron loss. Locus coeruleus hypopigmentation is associated with AD, vascular dementia, and FTD [37–39], whereas substantia nigra neuron loss is characteristic of LBD and other Parkinsonian syndromes [40, 41]. Taken together, global IIV related broadly to neuropathological outcomes associated with diverse neurodegenerative conditions. This high level of sensitivity in the context of limited specificity suggests global IIV may be particularly useful in the context of co-occurring neurodegenerative pathologies, which is the norm across neurodegenerative conditions [42, 43]. Future research should directly examine IIV associations with mixed pathologies.

In terms of neuropathological outcomes with domain-specific IIV, executive IIV was associated with AD (i.e., ADNC, CERAD, Braak) and cerebrovascular (i.e., CAA), memory IIV was associated with AD (i.e., CERAD, Braak), and language IIV was not significantly associated with any neuropathological outcome. The unique associations between memory IIV and AD outcomes suggest that episodic memory variability may reflect instability in networks specifically affected by neuritic plaque and NFT pathology, offering a behavioral correlate of early AD neural disruption. Higher memory IIV and executive IIV showed associations with greater neuritic plaque density and NFT severity, but not pure amyloid deposition (i.e., Thal phases), indicating potential specificity of these IIV metrics within ADRD pathology. Higher executive IIV also related to greater CAA severity, consistent with prior work linking mean-level executive function performance; [44, 45] however, replication is needed to confirm this relationship with IIV. The absence of significant associations between language IIV and neuropathological outcomes may reflect the limited sensitivity of language IIV to the specific neuropathological outcomes assessed, despite its relevance for long-term cognitive health. Notably, language IIV was associated with cognitive outcomes in the current study, suggesting that this dissociation is unlikely to reflect measurement unreliability. One possibility is that the language measures used to derive language IIV (e.g., naming and fluency) are sensitive to distributed, network-level dysfunction that impacts cognitive health but does not map cleanly onto the specific neuropathological outcomes examined here.

Global, executive, and memory IIV metrics were associated with neuropathological outcomes in cognitively unimpaired individuals, which is notable given autopsy data were available a mean of 8 years after the IIV metric was derived; however, the current study only modeled risk of progression to MCI/dementia and did not model cognitive trajectories between baseline and death, so it remains unclear whether IIV predicts future neuropathology or reflects preclinical disease processes already present. More research is needed to replicate IIV associations with neuropathological outcomes and disentangle prognostic value from its potential role as a marker of underlying preclinical neurodegeneration.

Contrary to the study hypothesis regarding sex differences, we found no significant sex-specific effects after FDR correction in either global or domain-specific IIV associations with cognitive status or neuropathological burden. Yet, we observed mean-level sex differences in global IIV, whichis consistent with past work showing higher global IIV in females relative to males [20–22]. The present work further refined early findings by showing domain-specific patterns of mean-level differences in IIV. Specifically, we found that language IIV was lower in females, memory IIV did not differ across sexes, and executive IIV was higher in females, although this difference was attenuated after adjustment for age, education, and race. Inconsistent with past work, the present analyses did not identify significant sex interactions, after correction for multiple comparisons, between IIV metrics and study outcomes. Differences in study focus may account for this discrepancy, given that previous investigations examined biological vitality and physical activity, whereas we evaluated longitudinal cognitive health and neuropathological burden [21, 23]. The current study extends past work by demonstrating the comparable utility across sexes of global and domain-specific IIV in relationships with cognitive status, cognitive diagnosis, progression to MCI/dementia, and neuropathological burden at autopsy.

### Limitations

Several important limitations should be considered in the context of the current study. Despite the large sample size, the current sample was largely White and highly educated, which may limit generalizability. Neuropathological assessments were only available for a subset of participants who were, unsurprisingly, older, disproportionately White, and more likely to have a dementia diagnosis at their final visit compared to those without available autopsy data. As such, the generalizability of these findings to the broader cohort may be limited. Additionally, NACC UDS is largely focused on ADRD, resulting in underrepresentation of other neurodegenerative disorders such as PPA and FTD which may show unique IIV associations. In vivo biomarkers of ADRD and neurodegeneration (e.g., CSF, plasma) were not examined within the current study, which represents a potential future direction. Although global and domain-specific IIV are relatively stable over time [15], we did not examine potential interactions between cognitive decline and IIV over time. Limitations of the cognitive battery determined the included scores and precluded the examination of other IIV domains (e.g., visuospatial). Future research using more comprehensive batteries should evaluate whether the observed relationships extend to other IIV domains. Relatedly, differences in scores used in the calculation of IIV and lack of consensus regarding its operationalization highlight the need for more standardization of IIV indices [46]. While differences in contributing scores primarily reflected structural variation across UDS versions rather than participant-level missingness, and domain-specific IIVs required at least two scores, version-related differences in test composition could nonetheless have influenced IIV estimates and should be considered in future work. Finally, although IIV has demonstrated incremental predictive value beyond mean-level cognitive performance, its comparative utility relative to traditional cognitive cutoffs and commonly used screening measures remains unclear and warrants direct comparison in future research [2]. 

### Conclusions

Study findings highlight the utility of IIV as an early indicator of longitudinal cognitive change and neuropathological burden that may help predict dementia risk. Findings replicated and extended past work by demonstrating global IIV is sensitive to long-term cognitive health and neuropathological burden spanning AD, cerebrovascular, and broader neurodegenerative etiologies. Domain-specific IIV was also associated with long-term cognitive outcomes, but demonstrated greater specificity in neuropathological outcomes, with memory IIV relating to AD outcomes and executive IIV relating to AD and cerebrovascular outcomes. Findings were largely consistent across sex, suggesting that associations of IIV with ADRD outcomes is similar among males and females. Together, IIV is a sensitive metric among cognitively unimpaired older adults that is robust to sex and may improve early risk stratification and inform tailored prevention efforts.

## Supplementary Information

Supplementary Material 1.

## Data Availability

The datasets analyzed during the current study are available as part of the National Alzheimer’s Coordinating Center Uniform Data Set to research investigators upon request, naccdata.org.

## Abbreviations

AD: Alzheimer’s disease
ADNC: Alzheimer’s disease neuropathologic change
ADRC: Alzheimer’s Disease Research Center
ADRD: Alzheimer’s disease and related dementias
BCFT: Benson Complex Figure Test
BNT: Boston Naming Test
Braak: Neurofibrillary tangle pathology severity
CAA: Cerebral amyloid angiopathy
CEARD: Neocortical neuritic plaque density
CIs: Confidence intervals
COV: Coefficient of variation
CST: Craft Story 21 Test
DS: Digit Span
DSST: Digit Symbol Substitution Test
FDR: False Discovery Rate Method
FTD: Frontotemporal dementia
FTLD-Tau: Frontotemporal lobar degeneration with tau pathology or other tauopathy
IIV: Intraindividual variability
IRB: Institutional review board
ISD: Intraindividual standard deviation
LBD: Lewy body disease
LM: Logical Memory
M: Mean
MCI: Mild cognitive impairment
MINT: Multilingual Naming Test
NACC: National Alzheimer’s Coordinating Center
NIA-AA: National Institute on Aging-Alzheimer’s Association
NFT: Neurofibrillary tangles
NST: Number Span Test
ORs: Odds ratios
PPA: Primary progressive aphasias
TMT: Trail Making Test
Thal: Thal phase for amyloid-β plaques
UDS: Uniform Data Set

## References

[1] Aita SL, Del Bene VA, Knapp DL, Demming CE, Ikonomou VC, Owen T, et al. Cognitive Intra-individual Variability in Cognitively Healthy APOE ε4 Carriers, Mild Cognitive Impairment, and Alzheimer’s Disease: a Meta-analysis. Neuropsychol Rev. 2024;21. 10.1007/s11065-024-09654-2. 10.1007/s11065-024-09654-2 39570562

[2] Chang F, Erani F, Landaverde D, Terao CM, Spence A, Zabala A, et al. Associations of cognitive intraindividual variability with Alzheimer’s disease risk: A systematic review. Clin Neuropsychol. 2025;1–36. 10.1080/13854046.2025.2547931. 10.1080/13854046.2025.2547931 PMC12871883 40847579

[3] Hultsch DF, MacDonald SWS, Hunter MA, Levy-Bencheton J, Strauss E. Intraindividual variability in cognitive performance in older adults: Comparison of adults with mild dementia, adults with arthritis, and healthy adults. Neuropsychology. 2000;14(4):588–98. 10.1037/0894-4105.14.4.588. 11055261 10.1037//0894-4105.14.4.588

[4] Stuss DT. Staying on the job: the frontal lobes control individual performance variability. Brain. 2003;126(11):2363–80. 10.1093/brain/awg237. 12876148 10.1093/brain/awg237

[5] Compton SE, Webber TA, Woods SP, Kiselica AM. Reliability and stability of cognitive intraindividual variability indices among cognitively unimpaired older adults. Clin Neuropsychol. 2025;1–19. 10.1080/13854046.2025.2552279. 10.1080/13854046.2025.2552279 PMC13330730 40944431

[6] Malek-Ahmadi M, Lu S, Chan Y, Perez SE, Chen K, Mufson EJ. Cognitive Domain Dispersion Association with Alzheimer’s Disease Pathology. Pahan K, editor. J Alzheimer’s Dis. 2017;58(2):575–83. 10.3233/JAD-161233 10.3233/JAD-161233 PMC6314665 28453479

[7] Jutten RJ, Amariglio RE, Maruff P, Properzi MJ, Rentz DM, Johnson KA, et al. Increased intraindividual variability in reaction time performance is associated with emerging cognitive decline in cognitively unimpaired adults. Neuropsychology. 2024;38(2):184–97. 10.1037/neu0000928. 37971861 10.1037/neu0000928 PMC12184789

[8] Bangen KJ, Weigand AJ, Thomas KR, Delano-Wood L, Clark LR, Eppig J, et al. Cognitive dispersion is a sensitive marker for early neurodegenerative changes and functional decline in nondemented older adults. Neuropsychology. 2019;33(5):599–608. 10.1037/neu0000532. 30896235 10.1037/neu0000532 PMC7380511

[9] Holmqvist SL, Thomas KR, Brenner EK, Edmonds EC, Calcetas A, Edwards L, et al. Longitudinal Intraindividual Cognitive Variability Is Associated With Reduction in Regional Cerebral Blood Flow Among Alzheimer’s Disease Biomarker-Positive Older Adults. Front Aging Neurosci. 2022;14:859873. 10.3389/fnagi.2022.859873. 35875798 10.3389/fnagi.2022.859873 PMC9300445

[10] Aschenbrenner AJ, Hassenstab J, Morris JC, Cruchaga C, Jackson JJ. Relationships between hourly cognitive variability and risk of Alzheimer’s disease revealed with mixed-effects location scale models. Neuropsychology. 2024;38(1):69–80. 10.1037/neu0000905. 37079810 10.1037/neu0000905 PMC10587364

[11] Gleason CE, Norton D, Anderson ED, Wahoske M, Washington DT, Umucu E, et al. Cognitive Variability Predicts Incident Alzheimer’s Disease and Mild Cognitive Impairment Comparable to a Cerebrospinal Fluid Biomarker. J Alzheimers Dis. 2017;61(1):79–89. 10.3233/JAD-170498. 10.3233/JAD-170498 PMC5714663 29125485

[12] Grewal KS, O’Connell ME, Kirk A, MacDonald SWS, Morgan D. Intraindividual variability measured with dispersion across diagnostic groups in a memory clinic sample. Appl Neuropsychol Adult. 2023;30(6):639–48. 10.1080/23279095.2021.1970552. 34455884 10.1080/23279095.2021.1970552

[13] Troyer AK, Vandermorris S, Murphy KJ. Intraindividual variability in performance on associative memory tasks is elevated in amnestic mild cognitive impairment. Neuropsychologia. 2016;90:110–6. 10.1016/j.neuropsychologia.2016.06.011. 27297728 10.1016/j.neuropsychologia.2016.06.011

[14] Scott BM, Austin T, Royall DR, Hilsabeck RC. Cognitive intraindividual variability as a potential biomarker for early detection of cognitive and functional decline. Neuropsychology. 2023;37(1):52–63. 10.1037/neu0000867. 36227289 10.1037/neu0000867

[15] DesRuisseaux LA, Guevara JE, Duff K. Examining the Stability and Predictive Utility of Across- and Within-Domain Intra-Individual Variability in Mild Cognitive Impairment. Arch Clin Neuropsychol. 2025;40(1):1–12. 10.1093/arclin/acae054. 39003237 10.1093/arclin/acae054 PMC12187063

[16] Kälin AM, Pflüger M, Gietl AF, Riese F, Jäncke L, Nitsch RM, et al. Intraindividual variability across cognitive tasks as a potential marker for prodromal Alzheimer’s disease. Front Aging Neurosci. 2014;6. 10.3389/fnagi.2014.00147. 10.3389/fnagi.2014.00147 PMC4081834 25071556

[17] Mascarenhas Fonseca L, Sage Chaytor N, Olufadi Y, Buchwald D, Galvin JE, Schmitter-Edgecombe M et al. Intraindividual Cognitive Variability and Magnetic Resonance Imaging in Aging American Indians: Data from the Strong Heart Study. Bangen K, editor. J Alzheimers Dis. 2023;91(4):1395–407. 10.3233/JAD-220825 10.3233/JAD-220825 PMC9974814 36641671

[18] Halliday DWR, Gawryluk JR, Garcia-Barrera MA, MacDonald SWS. White Matter Integrity Is Associated With Intraindividual Variability in Neuropsychological Test Performance in Healthy Older Adults. Front Hum Neurosci. 2019;13:352. 10.3389/fnhum.2019.00352. 31680907 10.3389/fnhum.2019.00352 PMC6803513

[19] Phillips M, Rogers P, Haworth J, Bayer A, Tales A. Intra-Individual Reaction Time Variability in Mild Cognitive Impairment and Alzheimer’s Disease: Gender, Processing Load and Speed Factors. Rypma B, editor. PLoS ONE. 2013;8(6):e65712. 10.1371/journal.pone.0065712. 10.1371/journal.pone.0065712 PMC3677873 23762413

[20] Merritt VC, Greenberg LS, Guty E, Bradson ML, Rabinowitz AR, Arnett PA. Beyond Measures of Central Tendency: Novel Methods to Examine Sex Differences in Neuropsychological Performance Following Sports-Related Concussion in Collegiate Athletes. J Int Neuropsychol Soc. 2019;25(10):1094–100. 10.1017/S1355617719000882. 31477193 10.1017/S1355617719000882

[21] Fagot D, Chicherio C, Albinet CT, André N, Audiffren M. The impact of physical activity and sex differences on intraindividual variability in inhibitory performance in older adults. Aging Neuropsychol Cogn. 2019;26(1):1–23. 10.1080/13825585.2017.1372357. 10.1080/13825585.2017.1372357 28868969

[22] Epstein JN, Karalunas SL, Tamm L, Dudley JA, Lynch JD, Altaye M, et al. Examining reaction time variability on the stop-signal task in the ABCD study. J Int Neuropsychol Soc. 2023;29(5):492–502. 10.1017/S1355617722000431. 36043323 10.1017/S1355617722000431 PMC9971352

[23] Watermeyer T, Massa F, Goerdten J, Stirland L, Johansson B, Muniz-Terrera G. Cognitive Dispersion Predicts Grip Strength Trajectories in Men but not Women in a Sample of the Oldest Old Without Dementia. Albert SM, editor. Innov Aging. 2021;5(3):igab025. 10.1093/geroni/igab025. 10.1093/geroni/igab025 PMC8448440 34549095

[24] Weintraub S, Besser L, Dodge HH, Teylan M, Ferris S, Goldstein FC, et al. Version 3 of the Alzheimer Disease Centers’ Neuropsychological Test Battery in the Uniform Data Set (UDS). Alzheimer Dis Assoc Disord. 2018;32(1):10–7. 10.1097/WAD.0000000000000223. 29240561 10.1097/WAD.0000000000000223 PMC5821520

[25] Besser LM, Kukull WA, Teylan MA, Bigio EH, Cairns NJ, Kofler JK, et al. The Revised National Alzheimer’s Coordinating Center’s Neuropathology Form—Available Data and New Analyses. J Neuropathol Exp Neurol. 2018;77(8):717–26. 10.1093/jnen/nly049. 29945202 10.1093/jnen/nly049 PMC6044344

[26] Jack CR, Bennett DA, Blennow K, Carrillo MC, Dunn B, Haeberlein SB, et al. NIA-AA Research Framework: Toward a biological definition of Alzheimer’s disease. Alzheimers Dement. 2018;14(4):535–62. 10.1016/j.jalz.2018.02.018. 29653606 10.1016/j.jalz.2018.02.018 PMC5958625

[27] Erani F, Terao CM, Cooper S, Weigand AJ, Bangen KJ, Edmonds EC, et al. Neuropsychiatric symptom phenotypes for early detection of risk in older adults. 2025. 10.1002/alz.70869. 10.1002/alz.70869 PMC12603784 41216845

[28] Montine TJ, Phelps CH, Beach TG, Bigio EH, Cairns NJ, Dickson DW, et al. National Institute on Aging–Alzheimer’s Association guidelines for the neuropathologic assessment of Alzheimer’s disease: a practical approach. Acta Neuropathol (Berl). 2012;123(1):1–11. 10.1007/s00401-011-0910-3. 22101365 10.1007/s00401-011-0910-3 PMC3268003

[29] Thal DR, Rüb U, Schultz C, Sassin I, Ghebremedhin E, Del Tredici K, et al. Sequence of Aβ-Protein Deposition in the Human Medial Temporal Lobe. J Neuropathol Exp Neurol. 2000;59(8):733–48. 10.1093/jnen/59.8.733. 10952063 10.1093/jnen/59.8.733

[30] Benjamini Y, Hochberg Y. Controlling the False Discovery Rate: A Practical and Powerful Approach to Multiple Testing. J R Stat Soc Ser B Stat Methodol. 1995;57(1):289–300. 10.1111/j.2517-6161.1995.tb02031.x.

[31] Holtzer R. Within-Person Across-Neuropsychological Test Variability and Incident Dementia. JAMA. 2008;300(7):823. 10.1001/jama.300.7.823. 18714062 10.1001/jama.300.7.823 PMC2736784

[32] Koscik RL, Berman SE, Clark LR, Mueller KD, Okonkwo OC, Gleason CE, et al. Intraindividual Cognitive Variability in Middle Age Predicts Cognitive Impairment 8–10 Years Later: Results from the Wisconsin Registry for Alzheimer’s Prevention. J Int Neuropsychol Soc. 2016;22(10):1016–25. 10.1017/S135561771600093X. 27903330 10.1017/S135561771600093X PMC5147731

[33] Tractenberg RE, Pietrzak RH. Intra-Individual Variability in Alzheimer’s Disease and Cognitive Aging: Definitions, Context, and Effect Sizes. Breitner JCS, editor. PLoS ONE. 2011;6(4):e16973. 10.1371/journal.pone.0016973. 10.1371/journal.pone.0016973 PMC3079725 21526188

[34] Duchek JM, Balota DA, Tse CS, Holtzman DM, Fagan AM, Goate AM. The utility of intraindividual variability in selective attention tasks as an early marker for Alzheimer’s disease. Neuropsychology. 2009;23(6):746–58. 10.1037/a0016583. 19899833 10.1037/a0016583 PMC2779520

[35] Patten RV, Fagan AM, Kaufman DAS. Differential Cued-Stroop Performance in Cognitively Asymptomatic Older Adults with Biomarker-Identified Risk for Alzheimer’s Disease: A Pilot Study. Curr Alzheimer Res. 2018;15(9):820–7. 10.2174/1567205015666180404170359. 29623843 10.2174/1567205015666180404170359

[36] Haynes BI, Bunce D, Kochan NA, Wen W, Brodaty H, Sachdev PS. Associations between reaction time measures and white matter hyperintensities in very old age. Neuropsychologia. 2017;96:249–55. 10.1016/j.neuropsychologia.2017.01.021. 28115193 10.1016/j.neuropsychologia.2017.01.021

[37] Arendt T, Brückner MK, Morawski M, Jäger C, Gertz HJ. Early neurone loss in Alzheimer’s disease: cortical or subcortical? Acta Neuropathol Commun. 2015;3(1):10. 10.1186/s40478-015-0187-1. 25853173 10.1186/s40478-015-0187-1 PMC4359478

[38] Freeze WM, Van Veluw SJ, Jansen WJ, Bennett DA, Jacobs HIL. Locus coeruleus pathology is associated with cerebral microangiopathy at autopsy. Alzheimers Dement. 2023;19(11):5023–35. 10.1002/alz.13096. 37095709 10.1002/alz.13096 PMC10593911

[39] Taheri S. Locus Coeruleus hypopigmentation in frontotemporal lobar degeneration and its relation to cerebrovascular injury biomarkers. Alzheimers Dement. 2023;19(S2):e061278. 10.1002/alz.061278.

[40] Kashihara K, Shinya T, Higaki F. Reduction of Neuromelanin-Positive Nigral Volume in Patients with MSA, PSP and CBD. Intern Med. 2011;50(16):1683–7. 10.2169/internalmedicine.50.5101. 21841326 10.2169/internalmedicine.50.5101

[41] Walker Z, Possin KL, Boeve BF, Aarsland D. Lewy body dementias. Lancet. 2015;386(10004):1683–97. 10.1016/S0140-6736(15)00462-6. 26595642 10.1016/S0140-6736(15)00462-6 PMC5792067

[42] Robinson JL, Xie SX, Baer DR, Suh E, Van Deerlin VM, Loh NJ, et al. Pathological combinations in neurodegenerative disease are heterogeneous and disease-associated. Brain. 2023;146(6):2557–69. 10.1093/brain/awad059. 36864661 10.1093/brain/awad059 PMC10232273

[43] Kapasi A, DeCarli C, Schneider JA. Impact of multiple pathologies on the threshold for clinically overt dementia. Acta Neuropathol (Berl). 2017;134(2):171–86. 10.1007/s00401-017-1717-7. 28488154 10.1007/s00401-017-1717-7 PMC5663642

[44] Planton M, Raposo N, Albucher JF, Pariente J. Cerebral amyloid angiopathy-related cognitive impairment: The search for a specific neuropsychological pattern. Rev Neurol (Paris). 2017;173(9):562–5. 10.1016/j.neurol.2017.09.006. 28993004 10.1016/j.neurol.2017.09.006

[45] Case NF, Charlton A, Zwiers A, Batool S, McCreary CR, Hogan DB, et al. Cerebral Amyloid Angiopathy Is Associated With Executive Dysfunction and Mild Cognitive Impairment. Stroke. 2016;47(8):2010–6. 10.1161/STROKEAHA.116.012999. 27338926 10.1161/STROKEAHA.116.012999

[46] Del Bene VA, Aita SL, Fonseca LM, Borgogna NC, Buchholz AS, Woods SP, et al. Cognitive intra-individual variability as an emerging measure of neuropsychological inference: A narrative review of its history, methodology, empirical support, future directions, and recommendations for best practices. Clin Neuropsychol. 2025;1–34. 10.1080/13854046.2025.2574463. 10.1080/13854046.2025.2574463 41118349

