# Supplementary material for: Global and domain-specific cognitive intraindividual variability associations with neurodegenerative diagnoses and postmortem pathologies
Source: Alzheimers Res Ther. 2026 Apr 21;18:143. doi: 10.1186/s13195-026-02041-4 (PMC13251277; doi:10.1186/s13195-026-02041-4)
Supplement: Supplementary file 1 — Supplementary Material 1. [file 13195_2026_2041_MOESM1_ESM.docx]

**Supplementary Material**

**Results**

**Associations of IIV with “Other Dementia” etiological clinical classification**

Due to low counts for Parkinson’s disease (*n*=6), atypical Parkinsonism syndromes (*n*=11), FTD (*n*=38), and PPA (*n*=4), these diagnostic categories as well as individuals with dementia who did not meet criteria for any of the listed major neurodegenerative conditions (*n*=67) were combined into an “other dementia” category (*n*=126).

Participants who at their last visit were classified as “other dementias” demonstrated greater variability in global IIV (OR=4.8, 95% CI: 2.5-8.9, *p*<.001) and language IIV (OR=24.6, 95% CI: 6.8-88.6, *p*<.001) relative to participants who remained cognitively unimpaired. There was no significant difference in memory IIV or executive IIV between cognitively unimpaired participants and those classified as other dementias.

**Supplementary Table 1**

*Sample Sizes for Cognitive Measures*

| Cognitive Measure | IIV Domain^1^ | *n* | % Sample |
| --- | --- | --- | --- |
| BCFT Copy | Global only | 8814 | 42.55 |
| BCFT Delayed Recall | Memory | 8799 | 42.48 |
| CST Immediate Paraphrased Recall | Memory | 8952 | 43.22 |
| CST Delayed Paraphrased Recall | Memory | 8948 | 43.20 |
| LM Immediate Recall | Memory | 11295 | 54.53 |
| LM Delayed Recall | Memory | 11296 | 54.53 |
| NST Forward | Executive | 9113 | 43.99 |
| NST Backward | Executive | 9108 | 43.97 |
| DS Forward | Executive | 11321 | 54.65 |
| DS Backward | Executive | 11321 | 54.65 |
| DSST | Executive | 10954 | 52.88 |
| TMT Part A | Executive | 20324 | 98.11 |
| TMT Part B | Executive | 20123 | 97.14 |
| BNT 30 | Language | 11284 | 54.47 |
| MINT | Language | 8826 | 42.61 |
| Semantic Fluency | Language | 20433 | 98.64 |
| Phonemic Fluency | Language | 8977 | 43.34 |

*Note.* LM = Logical Memory; BCFT = Benson Complex Figure Test; DS = Digit Span; BNT = Boston Naming Test; Multilingual Naming Test; NST = Number Span Test; DSST = Digit Symbol Substitution Test; CST = Craft Story 21 Test; TMT = Trail Making Test.

^1^ All scores were included in global IIV

**Supplementary Table 2**

*Sample Characteristics by Autopsy Subset*

|  | Autopsy Available  (*n*=1719) | Autopsy Not Available  (*n*=18996) | Test for Group Differences |
| --- | --- | --- | --- |
| Age^1^, *M*(*SD*) | 79.92 (8.25) | 69.81 (8.48) | *p*<.001 |
| Education, *M*(*SD*) | 15.83 (2.87) | 15.85 (2.96) | *p*=.808 |
| Race, *n* (%) |  |  | *p*<.001 |
| White | 1611 (93.72) | 14191 (74.71) |  |
| Black | 89 (5.18) | 3608 (18.99) |  |
| Asian | 9 (0.52) | 584 (3.07) |  |
| AIAN | 1 (0.06) | 211 (1.11) |  |
| NHPI | 1 (0.06) | 17 (0.09) |  |
| Other | 3 (0.17) | 255 (1.34) |  |
| Unknown | 5 (0.29) | 130 (0.68) |  |
| Final Diagnosis, *n* (%) |  |  | *p*<.001 |
| CU | 894 (52.01) | 16169 (85.12) |  |
| AD | 546 (31.76) | 1489 (7.84) |  |
| Impaired-not-MCI | 62 (3.61) | 513 (2.70) |  |
| MCI | 41 (2.39) | 412 (2.17) |  |
| Vascular | 87 (5.06) | 208 (1.09) |  |
| LBD | 45 (2.62) | 123 (0.65) |  |
| Other | 44 (2.56) | 82 (0.43) |  |
| IIV Global | 0.95 (0.29) | 0.88 (0.29) | *p*<.001 |
| IIV Language | 0.38 (0.14) | 0.37 (0.14) | *p*=.027 |
| IIV Executive | 0.97 (0.25) | 0.99 (0.24) | *p*=.028 |
| IIV Memory | 0.12 (0.12) | 0.17 (0.14) | *p*<.001 |

*Note.* AIAN = American Indian or Alaska Native; NHPI = Native Hawaiian or Other Pacific Islander; CU = cognitively unimpaired; AD = Alzheimer’s disease; MCI = mild cognitive impairment.

^1^ Age at baseline visit

**Supplementary Table 3**

*Sex by IIV Interaction Terms*

|  | OR | 95% CI | | *p* | *p_BH_* |
| --- | --- | --- | --- | --- | --- |
|  |  | LL | UL |  |  |
| ***Cognitive status*** (*n*=20,715) | | | | |  |
| Global IIV x Sex | 0.99 | 0.77 | 1.28 | .957 | .965 |
| Language IIV x Sex | 0.49 | 0.27 | 0.87 | .015 | .061 |
| Memory IIV x Sex | 1.01 | 0.62 | 1.64 | .965 | .965 |
| Executive IIV x Sex | 0.94 | 0.67 | 1.33 | .728 | .965 |
| ***ADNC*** (*n*=1232) |  |  |  |  |  |
| Global IIV x Sex | 1.10 | 0.48 | 2.50 | .822 | .822 |
| Language IIV x Sex | 0.69 | 0.14 | 3.30 | .641 | .822 |
| Memory IIV x Sex | 2.12 | 0.43 | 10.59 | .359 | .822 |
| Executive IIV x Sex | 0.71 | 0.28 | 1.81 | .472 | .822 |
| ***CERAD*** (*n*=1717) |  |  |  |  |  |
| Global IIV x Sex | 1.17 | 0.62 | 2.20 | .633 | .894 |
| Language IIV x Sex | 1.17 | 0.31 | 4.51 | .816 | .894 |
| Memory IIV x Sex | 1.10 | 0.27 | 4.51 | .890 | .894 |
| Executive IIV x Sex | 0.95 | 0.46 | 1.96 | .894 | .894 |
| ***Braak*** (*n*=1700) |  |  |  |  |  |
| Global IIV x Sex | 0.97 | 0.50 | 1.88 | .917 | .917 |
| Language IIV x Sex | 1.78 | 0.45 | 7.07 | .411 | .691 |
| Memory IIV x Sex | 5.43 | 1.23 | 24.08 | .026 | .102 |
| Executive IIV x Sex | 0.78 | 0.36 | 1.66 | .518 | .691 |
| ***Thal*** (*n*=1242) |  |  |  |  |  |
| Global IIV x Sex | 0.90 | 0.38 | 2.13 | .812 | .943 |
| Language IIV x Sex | 0.30 | 0.06 | 1.53 | .148 | .489 |
| Memory IIV x Sex | 1.06 | 0.19 | 5.89 | .943 | .943 |
| Executive IIV x Sex | 0.55 | 0.20 | 1.50 | .245 | .489 |
| ***Lewy Bodies*** (*n*=1714) | |  |  |  |  |
| Global IIV x Sex | 0.96 | 0.41 | 2.26 | .924 | .924 |
| Language IIV x Sex | 2.67 | 0.47 | 15.19 | .266 | .924 |
| Memory IIV x Sex | 0.87 | 0.14 | 5.43 | .881 | .924 |
| Executive IIV x Sex | 0.76 | 0.29 | 2.03 | .590 | .924 |
| ***Arteriolosclerosis*** (*n*=1542) | |  |  |  |  |
| Global IIV x Sex | 0.70 | 0.35 | 1.38 | .301 | . 603 |
| Language IIV x Sex | 1.16 | 0.28 | 4.76 | .842 | .842 |
| Memory IIV x Sex | 0.32 | 0.07 | 1.43 | .137 | .548 |
| Executive IIV x Sex | 0.88 | 0.40 | 1.96 | .762 | .842 |
| ***Atherosclerosis*** (*n*=1709) | |  |  |  |  |
| Global IIV x Sex | 0.85 | 0.45 | 1.62 | .623 | .831 |
| Language IIV x Sex | 1.48 | 0.39 | 5.56 | .563 | .831 |
| Memory IIV x Sex | 0.55 | 0.13 | 2.28 | .414 | .831 |
| Executive IIV x Sex | 0.96 | 0.46 | 2.02 | .919 | .919 |
| ***FTLD-Tau*** (*n*=1227) | |  |  |  |  |
| Global IIV x Sex | 1.15 | 0.36 | 3.71 | .810 | .962 |
| Language IIV x Sex | 0.95 | 0.10 | 8.55 | .962 | .962 |
| Memory IIV x Sex | 0.28 | 0.03 | 2.79 | .280 | .962 |
| Executive IIV x Sex | 1.36 | 0.35 | 5.23 | .658 | .962 |
| ***Cerebral Atrophy*** (*n*=1202) | |  |  |  |  |
| Global IIV x Sex | 0.93 | 0.39 | 2.23 | .879 | .959 |
| Language IIV x Sex | 2.78 | 0.54 | 14.36 | .220 | .882 |
| Memory IIV x Sex | 1.63 | 0.29 | 9.32 | .584 | .959 |
| Executive IIV x Sex | 0.97 | 0.36 | 2.64 | .959 | .959 |
| ***Hippocampal Atrophy*** (*n*=1210) | |  |  |  |  |
| Global IIV x Sex | 1.30 | 0.56 | 3.04 | .540 | .992 |
| Language IIV x Sex | 0.99 | 0.19 | 5.24 | .992 | .992 |
| Memory IIV x Sex | 0.78 | 0.15 | 4.15 | .776 | .992 |
| Executive IIV x Sex | 1.01 | 0.38 | 2.72 | .978 | .992 |
| ***Substantia Nigra Hypopigmentation*** (*n*=1230) | | |  |  |  |
| Global IIV x Sex | 1.76 | 0.69 | 4.58 | .241 | .591 |
| Language IIV x Sex | 1.14 | 0.19 | 7.05 | .885 | .885 |
| Memory IIV x Sex | 2.12 | 0.31 | 14.84 | .443 | .591 |
| Executive IIV x Sex | 1.56 | 0.54 | 4.52 | .411 | .591 |
| ***Locus Coeruleus Hypopigmentation*** (*n*=1184) | | |  |  |  |
| Global IIV x Sex | 1.35 | 0.55 | 3.31 | .515 | .719 |
| Language IIV x Sex | 0.87 | 0.15 | 4.85 | .870 | .870 |
| Memory IIV x Sex | 0.57 | 0.09 | 3.42 | .539 | .719 |
| Executive IIV x Sex | 1.76 | 0.62 | 5.02 | .291 | .719 |
| ***CAA*** (*n*=1694) |  |  |  |  |  |
| Global IIV x Sex | 0.88 | 0.45 | 1.73 | .716 | .955 |
| Language IIV x Sex | 0.99 | 0.25 | 3.84 | .983 | .983 |
| Memory IIV x Sex | 1.48 | 0.34 | 6.40 | .599 | .955 |
| Executive IIV x Sex | 0.72 | 0.33 | 1.56 | .402 | .955 |
| ***Infarcts and Lacunes*** (*n*=1714) | |  |  |  |  |
| Global IIV x Sex | 1.07 | 0.44 | 2.65 | .886 | .979 |
| Language IIV x Sex | 2.98 | 0.45 | 20.24 | .262 | .979 |
| Memory IIV x Sex | 1.28 | 0.16 | 10.72 | .817 | .979 |
| Executive IIV x Sex | 0.99 | 0.36 | 2.69 | .979 | .979 |
| ***Microinfarcts*** (*n*=1716) | |  |  |  |  |
| Global IIV x Sex | 1.51 | 0.66 | 3.46 | .329 | .465 |
| Language IIV x Sex | 3.35 | 0.62 | 18.16 | .159 | .465 |
| Memory IIV x Sex | 0.42 | 0.07 | 2.58 | .349 | .465 |
| Executive IIV x Sex | 1.27 | 0.50 | 3.23 | .617 | .617 |
| ***White Matter Rarefaction*** (*n*=1188) | | |  |  |  |
| Global IIV x Sex | 1.21 | 0.51 | 2.89 | .661 | .817 |
| Language IIV x Sex | 1.31 | 0.26 | 6.69 | .743 | .817 |
| Memory IIV x Sex | 1.22 | 0.23 | 6.52 | .817 | .817 |
| Executive IIV x Sex | 1.69 | 0.61 | 4.64 | .309 | .817 |
| ***Substantia Nigra Neuron Loss*** (*n*=1241) | | |  |  |  |
| Global IIV x Sex | 1.50 | 0.63 | 3.59 | .355 | .816 |
| Language IIV x Sex | 1.60 | 0.31 | 8.19 | .569 | .816 |
| Memory IIV x Sex | 1.43 | 0.26 | 7.87 | .681 | .816 |
| Executive IIV x Sex | 1.13 | 0.41 | 3.06 | .816 | .816 |

*Note.* OR = odds ratio; CI = confidence interval; LL = lower limit; UL = upper limit; ADNC = NIA-AA Alzheimer’s disease neuropathologic change; CERAD = neocortical neuritic plaque density; Braak = neurofibrillary tangle pathology severity; Thal = Thal phase for amyloid plaques; FTLD-Tau = frontotemporal lobar degeneration with tau pathology or other tauopathy; CAA = cerebral amyloid angiopathy.

**Supplementary Table 4**

*Sex Stratified Follow-Up Analyses*

|  | OR | 95% CI | | *p* |
| --- | --- | --- | --- | --- |
|  |  | LL | UL |  |
| ***Cognitive status and language IIV*** | | | | |
| Females | 6.98 | 2.51 | 19.64 | < .001 |
| Males | 1.31 | 0.44 | 3.96 | .626 |
| ***Braak and memory IIV*** |  |  |  |  |
| Females | 3.53 | 2.41 | 5.15 | < .001 |
| Males | 8.44 | 5.29 | 13.48 | < .001 |

*Note.* OR = odds ratio; CI = confidence interval; LL = lower limit; UL = upper limit; Braak = neurofibrillary tangle pathology severity.
